# Mindfulness on Daily Life Coping in People Experiencing Psychosis: A Randomized Controlled Trial

**DOI:** 10.1016/j.ijchp.2022.100298

**Published:** 2022-02-23

**Authors:** Emilio López-Navarro, Susana Al-Halabí

**Affiliations:** a Department of Psychology. University of Balearic Islands. Spain; b Department of Psychology. University of Oviedo. Spain

**Keywords:** Psychosis, Mindfulness, Coping, Randomized controlled trial, Experiment, psicosis, mindfulness, afrontamiento del estrés, ensayo clínico aleatorizado, experimento

## Abstract

**Background/Objective:**

Cognitive Behavior Therapy for psychosis (CBTp) is a recommended treatment for psychoses whose effect is mediated by coping. Mindfulness (MBI) have shown positive effects in psychosis. This study examines the hypothesis that combining CBTp+MBI could improve coping with day-to-day life in psychosis better than CBTp alone in people attending a public community rehabilitation center.

**Method:**

Fifty-six outpatients were recruited and randomly allocated either to CBTp or CBTp+MBI. Measures comprised PANSS interview and COPE Inventory. Data were analyzed using a repeated measures ANOVA and RCI calculation.

**Results:**

There were no statistical differences between groups at pre-treatment. Significant statistical differences were found for the interaction Treatment x Time in Mental disengagement (*F* = 5.65, *p* = .021, *η^2^* = .102), Acceptance (*F* = 7.69, *p* = .008, *η^2^* = .133), and Suppressing competing activities (*F* = 4.62, *p* = .037, *η^2^* = .085).

**Conclusions:**

MBI promotes specific coping styles in people who experience psychosis that otherwise are not improved with CBTp. Only the MBI group improved acceptance of the presence of the stressor and reduced mental disengagement from the context. The intervention is feasible and effective for public healthcare settings.

## Abstract (es)

**Antecedentes/Objetivo:**

La Terapia Cognitivo-Conductual para la psicosis (TCCp) es un tratamiento eficaz mediado por el afrontamiento del estrés. Las Intervenciones Basadas en Mindfulness (IBM) han demostrado efectos positivos en psicosis por lo que combinarlas con TCCp podría mejorar el afrontamiento. El estudio compara el efecto de la TCCp con TCCp+IBM sobre el afrontamiento en psicosis.

**Método:**

Cincuenta y seis participantes con psicosis fueron reclutados y asignados aleatoriamente a TCCp o TCCp+IBM. Los protocolos fueron sincronizados con las rutinas de los participantes. Las medidas incluyeron la entrevista PANSS y el inventario COPE. Los datos se analizaron con ANOVA de medidas repetidas y se calculó el RCI.

**Resultados:**

No hubo diferencias estadísticamente significativas entre grupos en el pre-tratamiento, sí las hubo en la interacción Tratamiento x Tiempo en desconexión mental (*F* = 5,65, *p* = 0,021, *η^2^* = 0,102), Aceptación (*F* = 7,69, *p* = 0,008, *η^2^* = 0,133), y Supresión de distractores (*F* = 4,62, *p* = 0,037, *η^2^* = 0,085).

**Conclusiones:**

La IBM fomenta estilos de afrontamiento en psicosis que la TCCp en solitario no. TCCp+IBM mejoró la aceptación del estresor y redujo la desconexión mental. La intervención es eficaz y viable en un contexto asistencial.

Psychotic experiences affect nearly 3% of people in developed countries and the social cost is estimated at 16,771 dollars per patient per year (Christensen et al., 2020; National Institute of Mental Health, 2021). There is significant debate about the proper way to refer to psychotic experiences. The different terms used reflect a more general debate about the nature and causes of these experiences, which are core features of the diagnostic systems available (American Psychiatric Association, 2013; World Health Organization, 2019). Defining entities such as psychotic disorder and schizophrenia is a tricky challenge because of the overlap between the clinical manifestations that define each entity, the heterogeneity of these manifestations in different people, and the way they change throughout life. Some people may have a psychotic experience on a single occasion, whereas others may experience them from time to time (for example during periods of stress) or frequently (Cooke, 2017). In order to streamline and agree on a common language between clinicians and researchers, psychotic experiences are classified into positive symptoms (i.e., experiences such as hearing voices or paranoia) and negative symptoms (i.e., diminished emotional expression or avolition).

Regardless of nomenclature, people experiencing psychosis show significant impairment in functional outcomes, including social and occupational functioning, independent living, and the ability to perform everyday activities (Al-Halabí et al., 2016). The aim of treatment is therefore twofold, as it focuses on reducing distress while recovering social roles (i.e., return to work or education) and preventing exacerbation. Hence, understanding the factors that may help to achieve both goals are key topics in clinical research in psychosis (NICE, 2020). In this regard, coping with stressors has been suggested as a variable that can improve treatment outcomes in psychosis (Gin et al., 2021).

Coping refers to the persons’ efforts to deal with events that outstrip their ability for an effective response (Folkman & Moskowitz, 2004). People experiencing psychosis tend to rely on avoidance and emotion-distraction coping (Piotrowski et al., 2020), and, as in other psychopathological phenomena, the experimental avoidance of the stressor results in an increase in psychotic experiences (Forman et al., 2021). Nevertheless, coping strategies focused on dealing with the stressor without avoiding it, while focusing on how to resolve it, have been related to reduced impact of psychotic experiences on well-being (López-Navarro et al., 2018), enhanced social functioning (Karaş et al., 2021), and reduction of the effect of stigma on recovery (Ordóñez-Camblor et al., 2021). Schlier et al. (2020) found that coping mediates the effect of Cognitive Behavior Therapy for psychoses (CBTp) on suspiciousness and negative symptoms. The NICE Guidelines recommend CBTp as a treatment for psychoses (Fonseca-Pedrero et al., 2021), and optimizing it is an ongoing challenge (Lysaker et al., 2020).

Mindfulness-based interventions (MBI) have demonstrated broad positive findings in people experiencing psychosis (Liu et al., 2021) as well other chronic mental disorders (Pardos-Gascón et al., 2021). These approaches involve noticing or becoming more aware of thoughts and experiences and accepting them as things that come and go, as thoughts rather than facts (Cooke, 2017). Mindfulness applied to psychosis teaches people to maintain contact with aversive psychotic experiences and react to them with acceptance instead of avoidance (Chadwick, 2019). MBI combined with CBTp improves the well-being and social functioning of people with psychosis (Chien et al., 2019; López-Navarro et al., 2015) and also reduces the intensity and frequency of negative symptoms (López-Navarro & Al-Halabí, 2021). Bearing in mind the mediating role of coping in the effectiveness of CBTp and the effects of mindfulness on psychosis, combining CBTp with MBI is a promising approach for optimizing treatment to improve coping in people experiencing psychosis (Jansen et al., 2020).

Based on the literature reviewed above, this study examines the hypothesis that combining CBTp and MBI could improve coping with day-to-day life in psychosis better than CBTp alone in people attending a public community rehabilitation center (ensuring the external validity of the study). To address this challenge, the study uses secondary data from a prior published RCT (López-Navarro et al., 2020).

## Method

### Design

A single center randomized controlled clinical trial with pre- and post-intervention measures was designed in a naturalistic clinical setting. The trial was registered in the ISRCTN Registry: ISRCTN52873519. Due the naturalistic features of the study and to avoid interfere with the community center routines, the sample size was based on service use of the later. Full details about the primary outcome can be consulted in López-Navarro et al. (2020) and effects on psychotic experiences in López-Navarro and Al-Halabí (2021).

### Participants

Participants were recruited from a community rehabilitation center in Spain. The study sample comprised a total of 52 randomized outpatients. The mean age of the sample was 39.71 years (*SD* = 8.98) with a mean duration of disorder of 14.13 years (*SD* = 7.66). Most of the participants were men (78.8%), and the mean number of years of education was 12.04 (*SD* = 2.08). The inclusion criteria were (1) aged between 18-65; (2) diagnoses that include presence of psychotic symptoms; (3) no changes in anti-psychotic drug treatment or hospitalization in the previous month; (4) signed informed consent; and (5) able to understand and read Spanish. Exclusion criteria were (1) significant cognitive impairment assessed through medical history or a medical condition that could bias the intervention outcome (e. g., dementia or cerebrovascular accident); (2) inability to attend mindfulness or rehabilitation treatment sessions; and (3) refusal to participate or to sign informed consent.

Participants were unemployed and received no remuneration for participating in the study. The study complied with the Declaration of Helsinki and was approved by the Research Ethics Committee of the University of Balearic Islands.

### Instruments and procedure

Clinical and demographic features of the sample were collected through a record form designed to cover age, sex, years since diagnosis, number of years of education, and clinical diagnosis. The latter was obtained through each participant's clinical record.

For descriptive purposes, the Spanish version of the Positive and Negative Syndrome Scale (PANSS; Kay et al., 1990) was used to assess the frequency and intensity of psychotic experiences in terms of positive and negative symptoms and general psychopathology. Each item is rated on a 7-point Likert scale (total scores ranging from 30 to 120). Higher scores indicate worse symptoms. To increase internal consistency, the interviews were videotaped and scored at the end of the intervention by two clinical psychologists blinded to participants’ allocation. Spanish version of the PANSS has shown adequate reliability indexes for positive (.72) and negative (.80) symptoms (Peralta & Cuesta, 1994).

The Coping Orientation to Problems Experienced (COPE; Carver et al., 1989) is a 60-item, multi-dimensional inventory developed to assess the different daily coping strategies people use in response to stress. The inventory is a list of statements that participants review and score. There are two main components to the COPE inventory: problem-focused coping and emotion-focused coping. Five scales aim to measure each of these: Problem-focused coping (Active coping, Planning, Suppression of competing activities, Restraint coping, and Seeking of instrumental social support); Emotion-focused coping (Seeking of emotional social support, Positive reinterpretation, Acceptance, Denial, and Turning to religion). It also contains three scales aimed at measuring coping responses: Focus on and venting of emotions, Behavioral disengagement, and Mental disengagement. The Spanish version of the COPE has showed a reliability above .75 for each factor except for Behavioral disengagement which is .53. The Spanish version of the COPE is considered equivalent to the original version ().

**Figure 1:**
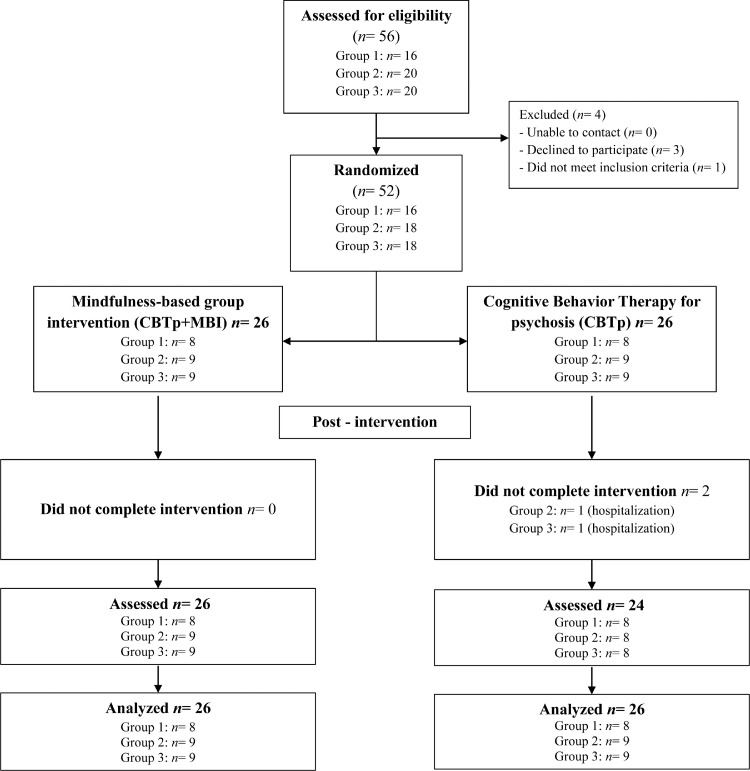
CONSORT flowchart.

To achieve strong external validity, the assessment and intervention protocols were naturally encompassed within participant's routines and the day-to-day functioning of a rehabilitation center belonging to the public health system. A clinical psychologist from the rehabilitation center contacted potential participants to schedule an interview to be informed about what participation in the trial would entail and to assess eligibility. Eligible participants were asked to participate. Once informed consent was signed, a randomization ID was assigned to each participant and recorded on the clinical record form. We created a master randomization list only accessible to the lead author and the clinical team that led the mindfulness sessions. Assessment of participants was conducted by a clinical psychologist who was blinded to the participants’ allocation. Once assessment was completed, the first author randomly allocated the participants by software to CBTp or CBTp+MBI, with a group size for MBI ranging from 8 to 12. Cohorts were randomized once there were enough participants to begin a mindfulness group. The recruitment process is detailed in Figure 1. Data collection was carried out at the same community rehabilitation center. Intention-to-treat analysis was used.

### Intervention

There were two treatment arms: CBTp and CBTp+MBI. Both interventions were delivered by a clinical psychologist from the rehabilitation center where the participants were recruited. Participants were under prior pharmacological treatment that was concurrent to the treatment arms in our study. No other intervention was concurrent to the treatment arms.

CBTp consisted of a 26-week rehabilitation treatment aimed at managing the intensity and frequency of psychotic symptoms, preventing relapses and hospitalization, as well as improving social functioning. In addition, CBTp entailed 26 weekly one-hour group sessions of cognitive behavior therapy for symptom management as well as strategies for conflict management and preventing relapse.

The CBTp+MBI group ran in parallel throughout the 26-week rehabilitation treatment program. MBI was implemented following the protocol described by López-Navarro et al. (2020). MBI aimed to teach participants to maintain contact with and accept the content of the psychotic experiences instead of avoiding or struggling with them or their content. According to the manual, MBI group sessions lasted 60 minutes, starting with a habituation period to the room with relaxing background music followed by 10-minute body awareness exercises led by a trained psychologist. This was followed by 15 minutes of guided meditation, encouraging awareness and acceptance of bodily sensations, sensations of breathing, and thoughts, images and voices that might occur. Finally, the sessions included 15 minutes of reflective group discussion aimed at facilitating patients’ understanding and insights drawn from the mindfulness practice. Home practice was encouraged between mindfulness sessions and participants were given an audio clip for home practice with the same guidance used during group sessions.

Detailed information covering CBTp intervention modules and content of mindfulness sessions are described in López-Navarro et al. (2020).

### Statistical analyses

Descriptive statistics were produced for the clinical and demographic features of the overall sample and separately for each treatment arm. Before conducting any analysis, assumptions of normality and homogeneity of variances were tested with Shapiro-Wilk and Levene tests. The primary outcome variable was daily-life coping measured via the scores in the fourteen scales in the COPE Inventory. Baseline comparisons were made between the groups in sociodemographic variables, psychotic symptoms, self-reported mindfulness, and the primary outcome variable. Intention-to-treat was used as an analytical strategy, thus, we used multiple stochastic imputation method to deal with missing data at post-treatment (Scharfstein & McDermott, 2019).

To test for group differences, a repeated-measures analysis of variance (ANOVA) was conducted for the COPE Inventory scores. Treatment condition (CBTp vs. CBTp + MBI) was set as the between-subjects factor and Time (pre- and post-treatment) as the within-subjects factor. This means that for every measure there are seven lines of results: the four simple effects, the two main effects - Treatment and Time-, and the interaction effect between these two factors. To analyze components of the interaction we used Bonferroni correction to control Type I errors across multiple comparisons. Eta squared was used for the effect size. If parametric assumptions were not met a bootstrapped ANOVA was performed at 2000 iterations. To estimate individual change in the primary outcome variable we used the Reliable Change Index (RCI) for those COPE scores where a statistically significant difference was found. RCI was computed according to the recommendations from Christensen and Mendoza (1986).

The data were analyzed with IBM SPSS 23 for Windows. Statistical significance was set at .05.

## Results

### Demographic and clinical features of the sample

Fifty-two participants were recruited in this study and randomly allocated to CBTp or CBTp+MBI groups. The CBTp and CBTp+MBI groups were compared in demographic and clinical variables, and we found no differences between the groups before treatment started. Demographic and clinical details are provided in Table 1. On average participants attended 91.58% of the 26 mindfulness sessions (range 21 to 26).

**Table 1:** Demographic and clinical features of the sample.

	Total sample(*N* = 52)	CBTp(*n* = 26)	CBTp + MBI (*n* = 26)	Statistics
Age (*M, SD*)	39.71 (8.98)	40.15 (9.38)	39.42 (8.63)	*t* = 0.29 *p* = .771
Sex (*n, %)*				*χ^2^* = 0.11 *p* = .734
Men	41 (78.8)	21 (80.8)	20 (76.9)	
Women	11 (21.2)	5 (19.2)	6 (23.1)	
Years since diagnosis (*M, SD*)	14.13 (7.66)	14.58 (8.22)	13.69 (7.2)	*t* = 0.41 *p* = .682
Education years (*M, SD*)	12.04 (2.08)	11.93 (1.89)	12.15 (2.25)	*t* = -0.39 *p* = .691
Diagnosis (*n, %)*				
Paranoid schizophrenia	23 (44.2)	11 (42.3)	12 (46.2)	*χ^2^* = 0.13 *p* = .999
Undifferentiated schizophrenia	8 (15.4)	4 (15.4)	8(15.4)	
Disorganized schizophrenia	4 (7.7)	2 (7.7)	2 (7.7)	
Schizoaffective disorder	11 (21.2)	6 (23.1)	5 (19.2)	
Bipolar disorder	4 (7.7)	2 (7.7)	2 (7.7)	
Delusional disorder	2 (3.8)	1 (3.8)	1 (3.8)	
PANSS (*M, SD*)				
Positive	14.9 (5.8)	14.69 (5.25)	15.11 (6.4)	*t* = -0.26 *p* = .796
Negative	19.08 (4.01)	19.16 (4.01)	19 (4.09)	*t* = 0.13 *p* = .892
General	36.11 (8.61)	36.5 (9.63)	35.74 (7.64)	*t* = 0.31 *p* = .751
Total	70.23 (15.46)	70.85 (15.25)	69.61 (15.94)	*t* = 0.28 *p* = .777

### Parametric assumptions analysis

Assessment of parametric assumptions of the pre-treatment COPE scores indicated that the scale scores that complied with the assumption of normality were Focus on and venting emotion*s, W*(52) = .96, *p* = .107; Seeking social support for instrumental reasons, *W*(52) = .98, *p* = .536; Restraint, *W*(52) = .97, *p* = .446; Seeking social support for emotional reasons, *W*(52) = .96, *p* = .088; and Planning, *W*(52) = .97, *p* = .208. At post-treatment the COPE scores that met the parametric assumptions were Seeking social support for instrumental reasons, *W*(52) = .96, *p* = .103, Acceptance, *W*(52) = .96, *p* = .08; and Suppressing competing activities, *W*(52) = .96, *p* = .075. Levene's test showed that at post-treatment COPE scores for Alcohol and drug disengagement did not comply with the assumption of homoscedasticity, *F*(50) = 5.54, *p* = .023. Therefore, the confidence intervals in the statistical analyses were bootstrapped.

### ANOVA on COPE scores: main factors and interaction

Repeated measures ANOVA on the COPE scores indicated no differences between groups at pre-treatment. In contrast, in the within-subjects factor we detected a statistically significant difference associated to a medium effect size for Mental disengagement, *F* = 4.57, *p* = .037, *η^2^*= .084; and to a large effect size for Focus on and venting emotions, *F* = 36.01, *p* < .001, *η^2^* = .419; Seeking social support for emotional reasons, *F* = 11.06, *p* = .002, *η^2^* = .181; Active coping, *F* = 15.13, *p* < .001, *η^2^*= .232; Denial, *F* = 81.74, *p* < .001, *η^2^* = .619; Restraint, *F* = 15.89, *p* < .001, *η^2^* = .241; Acceptance, *F* = 24.9, *p* < .001, *η^2^* = .333; Suppressing competing activities, *F* = 26.05, *p* < .001, *η^2^* = .343; and Planning, *F* = 16.2, *p* < .001, *η^2^* = .245. We did not find statistically significant differences in between-subjects factors in any COPE scores. Analysis of the interaction main effect revealed statistically significant differences associated to a medium effect size in Mental disengagement, *F* = 5.65, *p* = .021, *η^2^* = .102; Acceptance, *F* = 7.69, *p* = .008, *η^2^* = .133; and Suppressing competing activities, *F* = 4.62, *p* = .037, *η^2^* = .085. Table 2 provides detailed information regarding descriptive statistics and the ANOVA results from the COPE scores that were found to be statistically significant.

**Table 2:** Repeated Measures ANOVA on COPE scores where a statistically significant difference was found.

Outcome		Pretreatment(*M, SD*)	Posttreatment(*M, SD*)	*F*	*p* value	*Ƞ^2^*
Mental disengagement	CBTp	8.62 (1.67)	8.65 (1.29)	0.03	.867	.001
	CBTp+MBI	8.65 (1.72)	7.92 (1.23)	10.2	**.002**	**.169**
	Pre			0.01	.935	<.001
	Post			4.35	**.042**	**.08**
	Treatment			0.82	.369	.016
	Time			4.57	**.037**	**.084**
	Treatment x Time interaction			5.65	**.021**	**.102**
Focus on and venting emotions	CBTp	9.23 (1.61)	10.58 (1.84)	15.27	**<.001**	**.234**
	CBTp+MBI	9.27 (1.78)	10.85 (1.46)	20.96	**<.001**	**.295**
	Pre			0.01	.935	<.001
	Post			0.34	.561	.007
	Treatment			0.15	.7	.003
	Time			36.01	**<.001**	**.419**
	Treatment x Time interaction			0.22	.638	.004
Social support (emotional)	CBTp	9.92 (2.31)	10.92 (1.87)	6.77	**.012**	**.119**
	CBTp+MBI	10 (2.26)	10.81 (1.92)	4.42	**.041**	**.081**
	Pre			0.01	.905	<.001
	Post			0.05	.827	.001
	Treatment			0.01	.97	<.001
	Time			11.06	**.002**	**.181**
	Treatment x Time interaction			0.12	.725	.002
Active coping	CBTp	9.12 (1.92)	10.58 (1.94)	9.18	**.004**	**.155**
	CBTp+MBI	9.04 (1.51)	10.23 (1.97)	6.11	**.017**	**.109**
	Pre			0.03	.873	.001
	Post			0.41	.526	.008
	Treatment			0.31	.582	.006
	Time			15.13	**<.001**	**.232**
	Treatment x Time interaction			0.16	.582	.006
Denial	CBTp	10.08 (1.76)	7.85 (1.54)	28.46	**<.001**	**.363**
	CBTp+MBI	10.04 (1.75)	6.92 (1.41)	55.52	**<.001**	**.526**
	Pre			0.01	.937	<.001
	Post			5.07	**.029**	**.092**
	Treatment			1.99	.164	.038
	Time			81.74	**<.001**	**.619**
	Treatment x Time interaction			2.24	.141	.043
Restraint	CBTp	9.5 (2.52)	10.38 (1.6)	4.52	**.038**	**.083**
	CBTp+MBI	9.35 (2.43)	10.81 (1.47)	12.33	**.001**	**.198**
	Pre			0.05	.824	.001
	Post			0.98	.326	.019
	Treatment			0.08	.785	.002
	Time			15.89	**<.001**	**.241**
	Treatment x Time interaction			0.96	.332	.019
Acceptance	CBTp	10.42 (1.27)	10.96 (1.82)	2.46	.123	.047
	CBTp+MBI	10.19 (1.67)	12.08 (1.79)	30.16	**<.001**	**.376**
	Pre			0.31	.578	.006
	Post			4.97	**.03**	**.09**
	Treatment			1.29	.261	.025
	Time			24.9	**<.001**	**.333**
	Treatment x Time interaction			7.69	**.008**	**.133**
Suppressing competing activities	CBTp	10 (2.37)	10.85 (1.69)	4.36	**.042**	**.08**
	CBTp+MBI	10.08 (2.15)	12.15 (1.49)	26.3	**<.001**	**.345**
	Pre			0.01	.903	<.001
	Post			8.77	**.005**	**.149**
	Treatment			2.26	.139	.043
	Time			26.05	**<.001**	**.343**
	Treatment x Time interaction			4.62	**.037**	**.085**
Planning	CBTp	9.27 (1.99)	10.08 (1.35)	4.72	**.035**	**.086**
	CBTp+MBI	9.15 (2.29)	10.46 (2.27)	12.38	**.001**	**.198**
	Pre			0.04	.847	.001
	Post			0.55	.461	.011
	Treatment			0.07	.786	.001
	Time			16.2	**<.001**	**.245**
	Treatment x Time interaction			0.91	.346	.018

*Note.* CBTp and CBTp+MBI rows show interaction analysis for Time factor (Within subjects); Pre and Post rows show interaction analysis for Treatment factor (Between subjects)

Analysis of the interaction components indicated a statistically significant improvement for the CBTp group associated to a medium effect size for Seeking social support for emotional reasons, *F* = 6.77, *p* = .012, *η^2^* = .119; Restraint, *F* = 4.52, *p* = .038, *η^2^* = .083; Suppressing competing activities, *F* = 4.36, *p* = .042, *η^2^* = .08; and Planning scores, *F* = 4.72 *p* = .035, *η^2^* = .086; and to a large effect size for Focus on and venting emotions, *F* = 15.27, *p* < .001, *η^2^* = .234; Active coping, *F* = 9.18, *p* = .004, *η^2^* = .155; and Denial, *F* = 28.46, *p* < .001, *η^2^* = .363. In the CBTp+MBI group, we detected a statistically significant improvement associated to a medium effect size in Seeking social support for emotional reasons, *F* = 4.42, *p* = .041, *η^2^* = .081; Active coping, *F* = 6.11, *p* = .017, *η^2^* = .109; and to a large effect size in Mental disengagement, *F* = 10.2, *p* = .002, *η^2^* = .169; Focus on and venting emotions, *F* = 20.96, *p* < .001, *η^2^* = .295; Denial, *F* = 55.52, *p* < .001, *η^2^* = .526; Restraint, *F* = 12.33, *p* = .001, *η^2^* = .198; Acceptance, *F* = 30.16, *p* < .001, *η^2^* = .376; Suppressing competing activities, *F* = 26.3, *p* < .001, *η^2^* = .345; and Planning, *F* = 12.38, *p* = .001, *η^2^* = .198. When comparing treatment arms at post-treatment through analysis of the between-subjects component of the interaction we found that, compared to the CBTp group, the CBTp+MBI group exhibited a statistically significant reduction associated to a medium effect size in in Mental disengagement, *F* = 4.35, *p* = .042, *η^2^* = .08; and Denial scores, *F* = 5.07, *p* = .029, *η^2^* = .092; and a statistically significant increase associated to a medium effect size in Acceptance, *F* = 4.97, *p* = .03, *η^2^* = .09; and to a large effect size in Suppressing competing activities scores, *F* = 8.77, *p* = .005, *η^2^* = .149.

### Reliable Change Index calculation on COPE scores

RCI calculation was performed on those COPE scores that were found to be statistically significant in the ANOVA. For *Denial* scores, RCI estimation showed that 13 out of 26 in the CBTp group, and 22 out of 26 in the CBTp+MBI group presented a reliable change which was shown to be a significant difference between groups (*χ^2^* = 7.079, *p* = .008). In addition, for *Acceptance* scores, 9 out of 26 in the CBTp group exhibited a reliable change as did 16 out of 26 in the CBTp+MBI, which was statistically significant (*χ^2^* = 6.315, *p* = .012). Table 3 shows the RCI estimation and the comparison between treatment groups in the COPE scores that were found to be statistically significant in the repeated measures ANOVA.

**Table 3:** RCI calculation on COPE scores where a statistically significant difference was found in ANOVA.

COPE scores	Reliable Change Index				Statistics
	CBTp (*n, %*)		CBTp+MBI (*n, %*)		
	Yes	No	Yes	No	
Mental disengagement	5 (19.23)	21 (80.71)	6 (23.07)	20 (76.93)	*χ^2^* = 0.11 *p* = .734
Focus on and venting emotions	11 (42.31)	15 (57.69)	9 (34.61)	17 (65.39)	*χ^2^* = 0.32 *p* = .569
Social support (emotional)	7 (26.92)	19 (73.08)	8 (30.74)	18(69.23)	*χ^2^* = 0.94 *p* = .76
Active coping	18 (69.23)	8 (30.74)	12 (46.15)	14 (53.85)	*χ^2^* = 2.84 *p* = .092
Denial	13 (50)	13 (50)	22 (84.61)	4 (84.61)	***χ^2^* = 7.07** ***p* = .008**
Restraint	7 (26.92)	19 (73.08)	9 (34.61)	17 (65.39)	*χ^2^* = 0.36 *p* = .548
Acceptance	9 (34.61)	17 (65.39)	16 (61.54)	10 (38.46)	***χ^2^* = 6.31** ***p* = .012**
Suppressing competing activities	20 (76.93)	6 (23.07)	16 (61.54)	10 (38.46)	*χ^2^* = 1.44 *p* = .229

## Discussion

The main finding of our study is that MBI promotes specific coping styles in people who experience psychosis that otherwise are not improved with CBTp. Although both treatments improved coping styles associated with direct management of the behavioral efforts against stressors, the combination of CBTp+MBI led to greater improvements in the suppression of competing activities and reduced the use of denial strategies as pointed by the effect size registered. In fact, in line with our hypothesis, only the MBI group improved acceptance of the presence of the stressor and reduced behavioral disengagement from the context.

As expected, given the aim of CBTp, both treatment groups improved their coping based on give steps to resolve stressful situations and the use of emotional venting along with seeking emotional social support from other people. This finding is consistent with prior research pointing to mobilization of resources and venting of emotions as mechanisms of change in CBTp (Schlier et al., 2020). Nevertheless, although both groups exhibited decreased coping based on distraction and denial of the stressor, the group receiving mindfulness sessions exhibited a larger decrease as showed by the effect size detected. This effect may be accounted for by previously reported effects of mindfulness on attention and cognitive inhibition in psychosis (López-Navarro et al., 2020). As people who experience psychoses improve their cognitive control, it becomes easier to resist distractions, and in combination with acceptance, this may help them to not deny the existence of a source of distress (Lindsay & Creswell, 2019).

Participants in the CBTp+MBI group improved restraint and acceptance while reducing mental disengagement compared to those in the CBTp group. MBI applied to psychosis teaches people to maintain contact with the psychotic experience despite its content as well as accepting it like other mental phenomena. Participants in the MBI group generalized this attitude towards psychotic experiences to daily life stressors although they were not particularly trained to do so. This meant that people in the CBTp+MBI group learned to be aware of the stressor and not repel it from consciousness whilst preparing for the best time to make behavioral efforts to resolve the stressor. In other words, participants learned to accept that the stressor is real, despite the content, and wait for a proper set of contexts in which they could resolve it. Research has shown that, under laboratory conditions, people who experience psychosis can generalize learned behaviors from one context to another (Levin & Villatte, 2016). Our study extends this finding to adaptative behaviors taught in a real-world clinical context.

The clinical importance of the findings study is underscored by providing the first data about how CBTp in combination with mindfulness improves coping in people experiencing psychosis in real-world settings, which maximizes its external validity. More specifically, the combination of CBTp with MBI fosters behaviors—i.e., acceptance of the situation instead of denial—that are associated with better treatment outcomes and better mental health (Crits-Christoph & Connolly-Gibbons, 2021), and which have been indicated as mediators of the effect of negative symptoms on recovery (Chen et al., 2019). In addition, coping styles encouraged by CBTp+MBI have been associated with less stigma in psychosis (Prasko et al., 2016), which is a mediator of the effect of negative symptoms on recovery (Ordóñez-Camblor et al., 2021). These suggestive findings support the notion that MBI added to CBTp is a promising treatment for improving recovery from psychosis.

Our study has limitations and strengths that deserve mention. The limitations include an uneven gender distribution although it is in line with the gender distribution of psychosis; a small sample size (though it was enough to test the hypotheses); home practice of mindfulness was recommended but not assessed; the single center design used; and the CBTp group did not receive additional sessions to compensate for the extra training received by the CBTp+MBI group. The main strengths of the current study are the use of a randomized design with an active control condition; sustained mindfulness training assessed against the manual; a sample recruited from the same community center; and interventions delivered by routine clinical staff whilst being incorporated within participants’ daily routines, which increases the generalization of our results to daily-life practice of healthcare professionals treating psychoses.

Our findings add to the growing literature attesting for the safety and feasibility of mindfulness applied to psychosis. Our results go further and point to daily life coping as a mechanism of change of MBI that could help to improve day-to-day functioning. Future research should include a follow-up phase and assess the impact on service use due to improved coping through mindfulness training alone or in combination with psychological therapy. In addition, further research should explore how other cognitive variables, for example resilience (Pérez-Aranda et al., 2021) or transdiagnostic processes (Ramos et al., 2020), may mediate the effect of mindfulness in people experiencing psychosis. Further studies should also consider extending mindfulness to participants’ caregivers as it has beneficial effects for caregivers of people experiencing chronic disorders (Blanco-Donoso et al., 2021; Calvete et al., 2021; Strauss et al., 2021). It is important to note that mindfulness applied to persistent psychotic symptoms is a tool for developing a mindset against distress and suffering, not just a soothing exercise that has become mere fashion (Errasti-Pérez, Al-Halabí, López-Navarro, & Pérez-Álvarez).

In summary, our study provides the first data about the effects of combining mindfulness and CBTp on coping with daily stressors in a clinical setting within a public health system that aims to help people recover from psychosis.

## Funding

This work was supported by the European Social Fund-European Commission [Operative Project FSE 2014-2020]; the Board of Innovation, Research and Tourism of the Balearic Islands [FPI/1806/2015]; and the Spanish Ministry of Economy and Competitiveness [FFI2013-43270-P].
